# Post-traumatic Stress Disorder in Individuals With Autism Spectrum Disorder: A Literature Review

**DOI:** 10.7759/cureus.110949

**Published:** 2026-06-16

**Authors:** Alexandria Smith Martin, Floyd Thompson

**Affiliations:** 1 Research, Medical University of the Americas, Devens, USA; 2 Psychiatry, Eastern Oklahoma VAMC, Muskogee, USA

**Keywords:** autism spectrum disorder (asd), caregiver involvement, children, cognitive behavioral therapy (cbt), diagnosis, diagnostic overshadowing, eye movement desensitization and reprocessing (emdr), posttraumatic stress disorder (ptsd), trauma, trauma-informed care

## Abstract

This structured narrative literature review examined current evidence on the identification and management of post-traumatic stress disorder (PTSD) and trauma-related symptoms in children and adolescents with autism spectrum disorder (ASD). Literature was identified through searches of PubMed, PsycINFO, and Google Scholar, focusing on peer-reviewed English-language studies published between 2010 and 2025. Studies were included if they examined PTSD, trauma-related symptoms, assessment, diagnosis, or interventions in autistic children and adolescents, or provided an ASD-specific or broader neurodevelopmental context relevant to trauma. Findings were organized thematically due to heterogeneity in study designs, participant characteristics, diagnostic instruments, and outcome measures.

The reviewed literature suggests that PTSD and trauma-related symptoms may be frequently unrecognized in autistic youth due to assessment complexities related to symptom overlap, communication differences, sensory sensitivities, and diagnostic overshadowing. Adapted screening approaches, caregiver and educator input, and developmentally appropriate assessment strategies may improve recognition of trauma-related distress. Evidence for treatment remains limited, but modified trauma-focused interventions, including adapted cognitive behavioral therapy (CBT), eye movement desensitization and reprocessing, caregiver-assisted approaches, and trauma-informed care settings, may support more individualized management. Overall, current evidence supports the importance of early, ASD-sensitive recognition of trauma-related symptoms while highlighting the need for larger pediatric studies, validated screening tools, longitudinal follow-up, and greater diversity in study populations.

## Introduction and background

According to the Diagnostic and Statistical Manual of Mental Disorders, Fifth Edition (DSM-5), post-traumatic stress disorder (PTSD) is classified as a trauma- and stressor-related disorder that can develop after exposure to a traumatic event [1]. These traumatic events may include physical or sexual assault, witnessing or experiencing violence, near-death experiences, natural disasters, or other life-threatening situations. PTSD is commonly associated with intrusive memories, nightmares, avoidance, hypervigilance, exaggerated startle response, irritability, and emotional detachment. While the prevalence of PTSD in the overall pediatric population has been studied, its identification and clinical impact in children with autism spectrum disorder (ASD) remain less well understood. Large epidemiologic studies have described PTSD prevalence across general populations, but these findings may not fully capture the diagnostic challenges present in neurodevelopmental populations [2].

Recognizing PTSD in autistic children and adolescents can be difficult because trauma-related symptoms may overlap with core ASD characteristics. Social withdrawal, sleep disturbance, irritability, avoidance, emotional dysregulation, and repetitive behaviors may be interpreted as features of ASD rather than possible trauma-related responses. This diagnostic overshadowing may delay trauma-focused assessment and contribute to treatment approaches that address behavior without recognizing underlying distress [3]. Children with neurodevelopmental disorders may also experience or communicate trauma-related distress differently since communication differences, cognitive profiles, sensory sensitivities, and reliance on caregiver interpretation can affect symptom recognition [4]. Recent reviews have emphasized that PTSD assessment and treatment in autistic individuals remain underdeveloped, with limited validation of ASD-adapted screening tools and heterogeneous evidence regarding treatment [5].

The relationship between intellectual disability, ASD, trauma exposure, and behavioral symptoms further complicates assessment. Deb et al. described the use of psychotropic medications, including antipsychotics, among individuals with intellectual disabilities who exhibit aggressive behavior [3]. Although these medications may reduce behavioral symptoms, they may not address underlying trauma-related distress when trauma is unrecognized. Wigham et al. reported that individuals with intellectual disabilities experience a range of acute and chronic life stressors, including family separation, social isolation, bereavement, and repeated failure experiences [6]. These events may have a significant emotional impact, particularly when individuals have limited communication skills, difficulties with emotional regulation, or reduced access to supportive resources.

Assessment limitations also contribute to underrecognition. Wigham and Emerson reviewed trauma and life events in adults with intellectual disability and emphasized that language and cognitive differences, as well as reliance on caregiver or observational reports, may affect PTSD identification [7]. Even when caregiver reports or direct observations are used, internal symptoms such as intrusive memories, dissociation, or emotional numbing may still be missed. As a result, autistic children and adolescents may express trauma-related distress through behavior changes that are misunderstood as intentional behavioral problems or baseline ASD-related features.

Emerging ASD-focused research suggests that autistic individuals may have increased vulnerability to PTSD and trauma-related symptoms. Rumball et al. reported elevated PTSD symptoms in adults with ASD compared with individuals without ASD [8]. Although this study involved adults, the findings have developmental relevance because cognitive inflexibility, memory differences, sensory sensitivities, and limited coping strategies may also affect how autistic youth process and communicate trauma-related distress. These factors may make standard PTSD screening and treatment approaches less effective if they are not tailored to the developmental needs of autistic youth.

Many traditional trauma-focused interventions, including cognitive behavioral therapy (CBT), assume verbal expression, cognitive flexibility, and emotional insight. These assumptions may not fit all autistic children and adolescents, particularly those with communication differences, intellectual disability, alexithymia, or sensory sensitivities. Without developmentally appropriate assessment and intervention strategies, trauma-related distress may be missed or managed through approaches that do not fully address the child’s emotional needs. Collaboration among caregivers, educators, mental health professionals, and clinicians is therefore important for identifying changes from baseline functioning and supporting trauma-informed care.

Despite increasing recognition of trauma-related symptoms in autistic individuals, limited pediatric ASD-specific literature has examined how PTSD is identified, differentiated from core ASD features, and managed in children and adolescents. This structured narrative literature review examines current evidence on PTSD recognition, diagnostic challenges, assessment strategies, and trauma-informed interventions in autistic children and adolescents. The objective is to evaluate whether early and accurate identification of PTSD may improve trauma-related management and outcomes in this population.

## Review

Methods

This structured narrative literature review integrates current evidence on the identification and management of PTSD in children and adolescents under 18 years of age with ASD. 

PubMed, PsycINFO, and Google Scholar were searched for peer-reviewed English-language studies published between 2010 and 2025. This timeframe was selected to prioritize recent evidence on PTSD recognition, diagnostic tools, and trauma-informed interventions relevant to autistic children and adolescents. Search terms included combinations of “posttraumatic stress disorder,” “PTSD,” “autism spectrum disorder,” “ASD,” “autism,” “children,” “adolescents,” “pediatric,” “trauma,” “adverse events,” “assessment,” “diagnosis,” “intervention,” “cognitive behavioral therapy,” “CBT,” “eye movement desensitization and reprocessing,” and “EMDR.” Boolean operators were used to combine terms, including: (“PTSD” OR “posttraumatic stress disorder”) AND (“autism spectrum disorder” OR “ASD” OR “autism”) AND (“children” OR “adolescents” OR “pediatric”); (“trauma” OR “adverse events”) AND (“autism spectrum disorder” OR “ASD”) AND (“assessment” OR “diagnosis”); and (“autism” OR “ASD”) AND (“PTSD” OR “trauma”) AND (“intervention” OR “CBT” OR “EMDR”).

Studies were included if they were peer-reviewed, published in English between 2010 and 2025, and focused on PTSD, trauma-related symptoms, assessment, or treatment in children or adolescents with ASD. Studies were also considered if they provided relevant ASD-specific or neurodevelopmental context for diagnostic challenges, trauma presentation, or intervention adaptation. Studies were excluded if they were not peer-reviewed, did not involve human participants, focused only on adult populations without clear relevance to ASD or pediatric trauma, did not address PTSD or trauma-related symptoms, or were unavailable in full text.

After duplicate records were removed, titles and abstracts were screened for relevance to PTSD, trauma-related symptoms, ASD, and pediatric or neurodevelopmental populations. Full-text articles were then reviewed to determine whether they addressed PTSD identification, diagnostic challenges, trauma presentation, or intervention strategies relevant to autistic children and adolescents. Data were extracted using a standardized approach that captured the study category, author, population, study design, sample size, reported PTSD or trauma-related prevalence, assessment or diagnostic tool, key findings, and relevance to the review. Study limitations were also considered during manuscript synthesis.

The extracted information was organized into an evidence summary table (Table 1) and used to guide the narrative synthesis. The table allowed for comparison of similarities, differences, and emerging patterns across studies. This transparent approach supports reproducibility and may be repeated in future updates of the review. Given the heterogeneity of the included studies, including differences in study design, participant characteristics, diagnostic instruments, and outcome measures, a meta-analysis was not performed. Instead, findings were synthesized thematically. The main themes included PTSD prevalence and trauma-related symptom presentation, diagnostic challenges and screening limitations, and intervention approaches for autistic children and adolescents.

**Table 1:** Evidence summary of pediatric and contextual studies examining PTSD and trauma-related symptoms in ASD ASD: autism spectrum disorder; PTSD: post-traumatic stress disorder; ID: intellectual disability; ABA: applied behavior analysis; DSM-5: Diagnostic and Statistical Manual of Mental Disorders, Fifth Edition; N/A: not applicable

Category	Author(s)	Population	Study design	Sample size	Reported PTSD/trauma-related prevalence	Assessment/diagnostic tool	Key findings	Relevance to review
ASD-focused contextual evidence	Haruvi-Lamdan et al., 2020 [9]	Individuals with ASD compared with neurotypical controls	Cross-sectional study	25 ASD participants	32%	Self-reported PTSD questionnaire	PTSD symptoms were more frequently reported in individuals with ASD than in neurotypical controls, supporting the need for ASD-sensitive PTSD assessment	Provides ASD-specific evidence that PTSD symptoms may be elevated in autistic individuals
ASD-focused contextual evidence	Rumball, 2018 [10]	Individuals with ASD	Systematic review	N/A	N/A	Various PTSD assessment tools	PTSD assessment and treatment approaches varied widely across ASD studies, highlighting the need for validated ASD-adapted tools and interventions	Supports the need for clearer assessment and treatment approaches in ASD
Pediatric ASD-focused contextual evidence	Kildahl et al., 2019 [11]	Individuals with ASD and intellectual disability, including youth, are included in the studies	Systematic review	18 (studies)	40%	DSM-5 PTSD criteria, informant reports, and adapted assessment tools	PTSD symptoms were identified in ASD/ID populations, but standard PTSD tools were limited, particularly for individuals with communication impairment	Supports diagnostic challenges and the need for adapted PTSD assessment tools
Pediatric ASD-focused contextual evidence	Ng-Cordell et al., 2022 [12]	Children/adolescents with ASD	Cross-sectional study	102	40%	Caregiver reports	Trauma-related symptoms were reported in autistic youth, and caregiver input may improve recognition of trauma-related distress	Supports the need for trauma screening and caregiver-informed assessment in pediatric ASD
ASD-focused contextual evidence	Haruvi-Lamdan et al., 2018 [13]	Individuals with ASD	Literature review	N/A	N/A	DSM-5/PTSD literature synthesis	ASD and PTSD may co-occur, and symptom overlap can complicate diagnosis	Supports the conceptual framework for diagnostic overlap between ASD and PTSD
Broader neurodevelopment contextual evidence	Fernell and Gillberg, 2020 [14]	Individuals with borderline intellectual functioning and ASD-related concerns	Literature review	N/A	N/A	Literature synthesis	Borderline intellectual functioning commonly overlaps with neurodevelopmental conditions and may complicate psychiatric assessment	Provides background on cognitive/developmental factors that may influence PTSD recognition
Adult ASD contextual evidence	Kupferstein 2018 [15]	Autistic individuals exposed to ABA	Online survey	460	46%	Self-reported PTSD scale	PTSD symptoms were reported among some autistic individuals exposed to ABA, although findings were based on self-report and survey data	Provides contextual evidence that some intervention experiences may be associated with trauma-related symptoms in autistic individuals

Because this review was conducted as a structured narrative literature review, a formal risk-of-bias tool was not applied. Instead, studies were appraised narratively based on methodological clarity, relevance to pediatric ASD and trauma-related symptoms, population characteristics, assessment or diagnostic approaches, outcome measures, and stated limitations.

This review used previously published literature and did not involve human participants or identifiable patient data; therefore, institutional review board approval was not required. When available, ethical considerations reported in the included studies were noted during narrative appraisal, particularly for studies involving minors or individuals with developmental disabilities. This structured narrative approach was used to synthesize the available evidence while accounting for methodological variability and the limited amount of pediatric ASD-specific research in this field.

Results

The reviewed literature demonstrated substantial variability in study design, population characteristics, diagnostic methods, and treatment approaches. Despite this heterogeneity, three recurring themes emerged: possible increased trauma-related vulnerability and underrecognition among autistic children and adolescents, diagnostic challenges related to symptom overlap and limited ASD-adapted screening tools, and the need for individualized trauma-informed interventions. These themes are summarized below and supported by the evidence presented in Table 1.

PTSD Prevalence and Trauma-Related Symptom Presentation in Autistic Children

Available studies suggest that trauma-related symptoms and PTSD may be underrecognized in autistic children and adolescents; however, reported prevalence varies across studies because of differences in sample characteristics, diagnostic criteria, and assessment tools [9-12]. ASD-related communication differences, sensory sensitivities, and symptom overlap with PTSD may contribute to underdiagnosis or inconsistent reporting across clinical and research settings [10,11,13].

In ASD-focused studies, PTSD symptoms were reported more frequently among autistic individuals than in comparison groups, although many samples included adults or mixed-age populations [9,10]. Pediatric and neurodevelopmental studies also suggest that trauma-related distress may present through sleep disturbance, avoidance, irritability, regression, emotional dysregulation, or increased repetitive behaviors rather than through verbally reported intrusive memories alone [11,12]. These findings support the need for developmentally appropriate, ASD-sensitive trauma screening rather than reliance on standard PTSD presentations alone [10,11,13].

Diagnostic Challenges and Overshadowing

Across the reviewed literature, diagnostic overshadowing was a consistent concern. PTSD-related symptoms such as avoidance, emotional withdrawal, sleep disturbance, irritability, hyperarousal, and increased rigidity may be misattributed to core ASD characteristics rather than recognized as possible trauma-related responses [10,11,13]. This overlap can delay trauma-focused assessment and may lead clinicians to prioritize behavioral management without fully evaluating changes from the child’s baseline functioning.

Several studies also emphasized that standard PTSD screening tools may be limited for autistic children and adolescents, particularly for those with language impairment, intellectual disability, or difficulty describing internal emotional states [10,11,13]. As summarized in Table 2, caregiver reports, educator observations, adapted instruments, and developmentally appropriate interviewing strategies may improve recognition of trauma-related symptoms when self-report alone is insufficient.

**Table 2:** Diagnostic challenges and assessment considerations for PTSD in ASD populations PTSD: post-traumatic stress disorder; ASD: autism spectrum disorder; DSM-5: Diagnostic and Statistical Manual of Mental Disorders, Fifth Edition; IES-IDs: Impact of Event Scale – Intellectual Disabilities; N/A: not applicable

Author(s)	Sample size	Diagnostic tool(s)	Challenges in diagnosis	Key findings
					Rumball, 2018 [10]	N/A	Literature review, various PTSD tools	Lack of consistency in PTSD screening tools across ASD populations; most studies do not use ASD-specific diagnostic tools	Findings support the need for ASD-specific PTSD assessment tools because existing tools vary widely and are not consistently adapted for autistic individuals
Kildahl et al., 2019 [11]	18 (studies)	DSM-5 PTSD criteria, informant reports	Limited use of specialized assessment tools for ASD populations (e.g., IES-IDs)	PTSD symptoms can be identified in ASD/ID populations, but assessment is limited when individuals have reduced verbal communication
Haruvi-Lamdan et al., 2018 [13]	N/A	Self-reported PTSD questionnaire, DSM-5 criteria	Difficulty in distinguishing PTSD symptoms from ASD characteristics (e.g., social withdrawal, rigidity)	Trauma-related symptoms in ASD may present atypically, increasing the risk of diagnostic overshadowing
Kupferstein, 2018 [15]	460	Self-reported PTSD scale, online survey	Difficulty in self-reporting PTSD symptoms, especially in individuals with limited verbal skills or communication barriers	Self-report findings suggest trauma-related symptoms may occur in autistic individuals, but communication differences and reporting bias should be considered

These findings highlight the importance of combining caregiver input, developmental context, and ASD-adapted screening methods when evaluating trauma-related symptoms in autistic children and adolescents.

Trauma Screening Tools and Measurement Strategies

The reviewed literature suggests that PTSD screening in autistic children should not rely solely on standard self-report tools. Communication differences, alexithymia, intellectual disability, and difficulty identifying internal emotional states may limit the accuracy of traditional PTSD measures [10,11,13]. One study reported limited routine trauma screening in child psychiatry settings, despite behavioral decline being a possible sign of trauma-related symptoms in children with ASD [11]. Adapted approaches, including caregiver-informed assessments, educator observations, visual supports, and developmentally appropriate interview techniques, may improve recognition of trauma-related symptoms in this population [10-12].

However, the evidence remains limited, and few ASD-adapted PTSD screening tools have been validated across diverse pediatric samples. This limits the ability to compare findings across studies and reinforces the need for standardized, developmentally appropriate screening strategies for autistic children and adolescents.

Therapeutic Interventions and Treatment Outcomes

Evidence on PTSD treatment in autistic children and adolescents remains limited, but available studies suggest that trauma-focused interventions may be more effective when adapted to the child’s developmental, sensory, and communication needs [10,12]. Modified CBT approaches may require visual supports, simplified language, caregiver involvement, flexible pacing, and attention to sensory sensitivities [10,12]. Some studies also suggest that caregiver involvement may support symptom recognition, regulation, and treatment engagement, particularly when caregivers help identify triggers and reinforce coping strategies outside of therapy [10,12].

Other trauma-focused approaches, including eye movement desensitization and reprocessing (EMDR), have also been discussed as potentially useful for autistic individuals who have difficulty verbally describing traumatic experiences [10]. However, the evidence base remains small, and many studies lack long-term follow-up, diverse samples, or controlled designs. Overall, the literature supports individualized, trauma-informed intervention planning rather than a one-size-fits-all treatment model [10,12].

Patterns, Gaps, and Cross-Study Insights

Across studies, several limitations were consistent. Most studies included small samples, mixed pediatric and adult populations, variable PTSD definitions, inconsistent outcome measures, and limited longitudinal follow-up. Few studies used randomized or controlled designs, and limited data were available on racially, culturally, linguistically, or socioeconomically diverse populations [9-13]. These limitations restricted direct comparison across studies and limited the strength of conclusions that could be drawn.

Despite these limitations, the reviewed literature consistently supported the importance of considering trauma-related symptoms when autistic children and adolescents demonstrate new or worsening changes in sleep, behavior, emotional regulation, avoidance, or functional participation. The findings also emphasized the role of caregiver and educator input, ASD-adapted assessment strategies, and individualized trauma-informed interventions. Although available evidence suggests that developmentally appropriate and communication-sensitive approaches may improve recognition and treatment planning, additional studies are needed to determine whether early PTSD identification leads to sustained improvements in long-term outcomes.

Discussion

*Interpretation of Findings* 

This review highlights the clinical challenge of recognizing PTSD and trauma-related symptoms in autistic children and adolescents. Across the reviewed literature, trauma-related distress was often difficult to identify because symptoms such as sleep disturbance, irritability, avoidance, emotional withdrawal, regression, and increased rigidity may overlap with or be misattributed to core ASD characteristics [9-13]. This supports the concern that diagnostic overshadowing may delay trauma-focused assessment and intervention in this population.

The findings suggest that PTSD in autistic children and adolescents may not always present through verbally reported intrusive memories or avoidance of clearly identified traumatic events. Instead, trauma-related symptoms may appear as changes in baseline behavior, sensory tolerance, emotional regulation, sleep, rigidity, or functional participation. Because these changes may be interpreted as part of ASD rather than possible trauma-related distress, clinicians may miss opportunities for earlier trauma-focused evaluation. This interpretation should be made cautiously, however, because the available evidence remains limited by small samples, heterogeneous populations, and inconsistent diagnostic methods.

The reviewed literature also suggests that autistic children and adolescents may experience significant distress from both DSM-5-defined traumatic events and chronic adverse experiences, such as bullying, social exclusion, repeated sensory overload, unsupported academic demands, or interventions that are not adapted to sensory and emotional needs [12,13,15]. Although these chronic stressors may not always meet formal DSM-5 Criterion A for PTSD, they may still contribute to trauma-related symptoms, functional decline, or emotional dysregulation in autistic children and adolescents. For this reason, clinicians should distinguish a formal PTSD diagnosis from broader trauma-related distress while still assessing both carefully.

The findings also emphasize the need for developmentally appropriate and ASD-sensitive assessment strategies. Standard PTSD screening tools may miss trauma-related symptoms in autistic children and adolescents who have difficulty with verbal expression, abstract emotional language, or identifying internal distress. Caregiver and educator observations may therefore be important, particularly when symptoms are expressed through behavioral change, sleep disruption, avoidance, regression, or altered sensory tolerance [10,11,13]. However, these reports should be interpreted alongside direct clinical assessment when possible, because informant reports may not fully capture internal symptoms such as intrusive memories, dissociation, or emotional numbing [10,11,13].

Although evidence for PTSD treatment in autistic children and adolescents remains limited, the reviewed literature suggests that trauma-focused interventions may be more clinically useful when adapted to the child’s developmental, sensory, and communication needs. Rather than applying standard protocols without modification, clinicians may need to use visual supports, simplified language, flexible pacing, caregiver participation, and sensory-sensitive environments [10,12]. These adaptations may improve engagement and reduce the risk of overwhelming the child during trauma-focused work.

Caregiver involvement also appears clinically important because caregivers may recognize changes in baseline functioning, help identify triggers, and reinforce regulation strategies outside the therapy setting [12]. Educators may also provide valuable insights because trauma-related symptoms may appear in school as avoidance, withdrawal, irritability, behavioral escalation, or reduced participation. However, caregiver and educator reports should be balanced with direct clinical assessment when possible, and future studies should evaluate which caregiver-assisted and school-based adaptations are most effective across different ages, communication abilities, and ASD severity levels.

The findings also suggest that trauma-informed care should extend beyond individual therapy. Schools, clinics, and behavioral health programs should consider whether new or worsening behaviors may reflect trauma-related distress rather than assuming they represent only core ASD symptoms or noncompliance. This is especially important when interventions are intensive, behavior-focused, or insufficiently adapted to the child’s sensory and emotional needs [12,15]. A trauma-informed approach may help reduce misinterpretation of behavior and support more individualized treatment planning.

Overall, the reviewed literature suggests that PTSD and trauma-related symptoms in autistic children and adolescents may be missed when clinicians rely only on standard PTSD presentations or interpret behavioral changes solely through an ASD framework. Although the evidence remains limited, these findings support the need for trauma-informed assessment, ASD-adapted screening strategies, caregiver and educator input, and individualized intervention planning across clinical, school, and behavioral health settings.

*Impact on the Hypothesis* 

The findings of this review are generally consistent with the hypothesis that early and accurate identification of PTSD may improve trauma-related management in autistic children and adolescents. Across the reviewed literature, recognition of trauma-related symptoms appeared to shift clinical attention from general behavioral management toward more individualized, trauma-informed assessment and intervention strategies [9-13].

This is clinically important because symptoms such as aggression, regression, avoidance, sleep disturbance, emotional dysregulation, or withdrawal may otherwise be misattributed to core ASD characteristics rather than evaluated as possible trauma-related responses. Available evidence suggests that adapted approaches, including ASD-sensitive assessment strategies, modified trauma-focused interventions, and caregiver involvement, may improve engagement and guide more appropriate treatment planning [10-12].

However, the strength of this conclusion is limited by the small number of pediatric ASD-specific studies, heterogeneous diagnostic methods, and limited long-term follow-up. Stronger prospective studies are needed to determine whether early PTSD identification leads to sustained improvements in long-term outcomes such as emotional regulation, sleep, social functioning, quality of life, and reduced psychiatric comorbidity.

Limitations in the evidence

The findings of this review should be interpreted in light of several limitations in the available literature. Although increasing attention has been given to trauma and PTSD in autistic children and adolescents, the evidence base remains limited. Many included studies had small sample sizes, mixed pediatric and adult populations, or focused on broader neurodevelopmental or intellectual disability populations rather than pediatric ASD specifically. These factors limit statistical strength, restrict subgroup analysis, and reduce the generalizability of findings to autistic children and adolescents.

Additional limitations include inconsistent definitions of trauma, variable use of PTSD diagnostic criteria, limited validation of ASD-adapted screening tools, and short or absent longitudinal follow-up. Some studies focused on DSM-5-defined traumatic events, whereas others considered broader chronic stressors, sensory overload, bullying, or social exclusion as potentially trauma-related experiences. Although this broader view may be clinically relevant for autistic youth, it also makes it difficult to compare prevalence rates, treatment response, and long-term outcomes across studies.

The available literature also included limited racial, linguistic, socioeconomic, and cultural diversity, which restricts the ability to apply findings across varied clinical, school, and community settings. In addition, few studies appeared to include autistic individuals, caregivers, or families in the development or evaluation of screening tools and interventions, which may limit real-world relevance and acceptability.

Despite these limitations, the literature consistently points to the need for careful assessment of trauma-related symptoms when autistic children and adolescents show new or worsening behavioral, emotional, sleep-related, or functional changes. These limitations highlight the need for stronger pediatric ASD-specific research before firm conclusions can be made about prevalence, screening accuracy, and long-term treatment outcomes.

Future research directions

Future research should focus on developing and validating PTSD screening tools specifically adapted for autistic children and adolescents. These tools should account for communication differences, sensory sensitivities, intellectual disability, alexithymia, caregiver observations, nonverbal or behavioral expressions of distress, and changes from baseline functioning. Standardized assessment strategies would improve diagnostic consistency and allow better comparison across studies.

Additional longitudinal and prospective studies are needed to determine whether early PTSD recognition improves long-term outcomes such as emotional regulation, sleep, school participation, social functioning, quality of life, resilience, and reduced psychiatric comorbidity. Future studies should also evaluate how autistic children and adolescents respond to trauma-focused interventions over time, including whether early identification changes outcomes into adolescence and adulthood.

Future work should evaluate adapted trauma-focused interventions, including modified CBT, EMDR, caregiver-assisted approaches, teacher education, and school-based trauma-informed supports. Because many existing studies were conducted in specialized or highly resourced settings, research should examine whether these approaches can be implemented effectively in community clinics, schools, and other real-world care environments.

Finally, future research should include more racially, culturally, linguistically, and socioeconomically diverse populations. Participatory research involving autistic individuals, families, caregivers, educators, and clinicians may help ensure that future screening tools and interventions are clinically useful, developmentally appropriate, accessible, and responsive to the lived experiences of autistic youth.

## Conclusions

Autistic children and adolescents may be at increased risk for PTSD and trauma-related symptoms that are underrecognized because trauma responses can overlap with core ASD characteristics, communication differences, sensory sensitivities, and changes in baseline functioning. The reviewed literature suggests that trauma-related distress may present through regression, withdrawal, sleep disturbance, emotional dysregulation, avoidance, sensory overload, or other behavioral changes rather than through verbally reported symptoms alone. ASD-adapted assessment strategies, caregiver and educator input, and individualized trauma-informed interventions may improve recognition and management of trauma-related distress in this population.

Modified screening approaches, developmentally appropriate interviewing strategies, and trauma-focused interventions such as adapted CBT or EMDR may be useful when tailored to the child’s developmental, sensory, and communication needs. However, the available evidence remains limited by small sample sizes, inconsistent PTSD definitions, variable diagnostic tools, limited longitudinal follow-up, and the underrepresentation of diverse populations. Future research should prioritize validated pediatric ASD-specific PTSD screening tools, prospective outcome studies, and accessible trauma-informed interventions across clinical, school, and community settings. Collaboration among clinicians, educators, caregivers, researchers, and policymakers will be important to reduce diagnostic gaps and support timely, compassionate, and individualized care for autistic youth.
